# Unveiling the dual role of circulating tumor cells in colorectal cancer immunotherapy: a comprehensive review of biomarker utility and immune microenvironment crosstalk

**DOI:** 10.3389/fimmu.2025.1591359

**Published:** 2025-06-06

**Authors:** Yifan Feng, Gang Liu, Qixue Cai, Jianping Zhou

**Affiliations:** ^1^ Department of Gastrointestinal Surgery, The First Hospital of China Medical University, Shenyang, Liaoning, China; ^2^ Shenyang Medical Nutrition Clinical Medical Research Center, Shenyang, Liaoning, China; ^3^ Department of Pulmonary and Critical Care Medicine, Institute of Respiratory Disease, The First Hospital of China Medical University, Shenyang, Liaoning, China

**Keywords:** circulating tumor cell, colorectal cancer, microsatellite instability (MSI), immunotherapy, drug resistance, immune microenvironment

## Abstract

Colorectal cancer (CRC) has the highest incidence in the Asia-Pacific region, accounting for 51.8% of global cases. Despite early screening methods like colonoscopy, CT, and MRI, 20-25% of patients are diagnosed at advanced stages, with some having liver metastasis. Personalized treatments, including targeted and immunotherapy, are crucial for metastatic or recurrent CRC. Circulating tumor cells (CTC), emerging as a non-invasive biomarker, play a key role in assessing metastasis and prognosis. CTC count is linked to CRC stage, microsatellite instability (MSI-H), and drug resistance, and is valuable in evaluating the response to immune checkpoint inhibitors (ICIs). Immune cells in the tumor microenvironment influence CTC behavior, impacting metastasis, immune evasion, and resistance. Advances in CTC detection and genetic markers offer new possibilities for early diagnosis and personalized treatment.

## 1 Introduction

Colorectal cancer (CRC) incidence is the highest in the Asia-Pacific region, accounting for 51.8% of the global burden. With a growing population, a high Human Development Index (HDI), and rapid economic growth, the region faces an increasing challenge (1). Early screening methods, including colonoscopy, abdominal computed tomography (CT), and magnetic resonance imaging (MRI), can reduce the risk of colorectal cancer (2–4). Approximately 20-25% of patients are diagnosed at stage IV during their initial examination, with 10-15% presenting with colorectal cancer liver metastasis (CRCLM) (5). Additionally, among patients undergoing curative surgery for CRC, about 40% experience recurrence, primarily in the form of either local or distant metastasis (6, 7). For patients with metastatic or recurrent CRC, personalized treatment options, such as targeted therapy or immunotherapy, are essential for preventive treatment (8, 9). Biomarkers to guide the selection of the most appropriate therapy include tumor histology, such as KRAS/BRAF mutations, HER2 amplification, and microsatellite instability-high (MSI-H). Previous assessments were based on postoperative pathology. In contrast, liquid biopsy (LB) and circulating tumor cell (CTC) assessment offer a non-invasive and easily accessible technique that can improve personalized treatment before surgery (10).

Circulating tumor cells (CTCs) are emerging tumor biomarkers, referring to somatic cells that detach from the primary tumor and migrate into the circulatory system, which can lead to liver metastasis via the hepatic portal vein (11). CTC count has been established as an independent prognostic factor for patients with metastatic CRC (12). CTCs were first discovered by Thomas Ashworth in 1869, but it was not until the 1970s, with the rapid development of molecular biology technologies, that the enrichment and characterization of CTCs became feasible (13). Furthermore, the NCCN guidelines recognize the importance of CTCs in preoperative screening for central nervous system cancers, as well as in guiding treatment decisions for advanced prostate and breast cancers (14–17).

**Figure 1:**
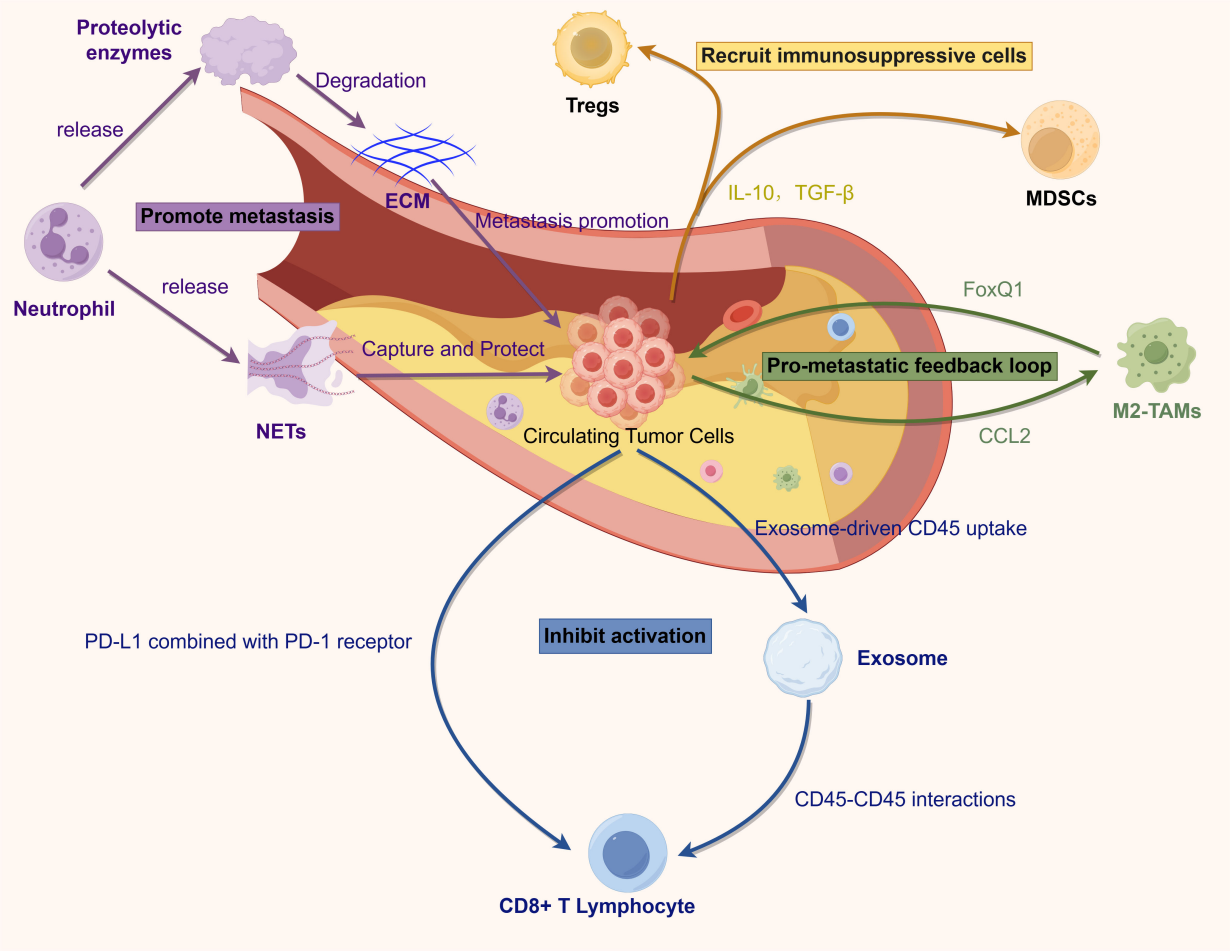
Shed tumor cells in peripheral blood can evade immune surveillance and immune killing through various mechanisms. ECM, extracellular matrix; NETs, neutrophil extracellular traps; Tregs: regulatory T cells; MDSCs, myeloid derived suppressor cells; IL-10, interleukin-10; TGF-β, Transforming Growth Factor-β; FoxQ1, forkhead box Q1; CCL2, C–C motif chemokine ligand 2; M2-polarized tumor-associated macrophages; PD-L1, programmed death-ligand 1; PD-1, programmed cell death protein 1.

Immunotherapy for colorectal cancer (CRC) works by blocking immune checkpoint (IC) pathways. Cancer cells can disguise themselves as normal cells through the IC pathways (**Figure 1**) (18). Tumor cells can express inhibitory ligands such as cytotoxic T-lymphocyte-associated protein 4 (CTLA-4) and programmed cell death ligand 1 (PD-L1), which send “stop” signals to active T cells, enabling the tumor to escape cell-mediated immunity. These studies have driven medical advancements, ushering in the era of precision medicine. The development of monoclonal antibodies targeting PD-1 (nivolumab and pembrolizumab) and PD-L1 (durvalumab and atezolizumab) has made enhanced antitumor immunity possible. Immunotherapy can improve clinical outcomes and extend overall survival (OS) (19, 20). However, only a small subset of CRC patients can benefit from immune checkpoint therapy (ICT) (21). Only those with MSI-H or defective mismatch repair (dMMR) in their CRC tumors demonstrate favorable treatment responses (22). This is due to insertions or deletions of nucleotides that can lead to DNA or microsatellite repeats. The accumulation of these mutations results in the generation of novel neoantigens, which can be recognized by the host immune system (20). Therefore, preoperative screening to identify patients who are suitable for immune checkpoint inhibitors (ICIs) treatment is crucial for the management of advanced colorectal cancer.

## 2 Clinical significance of circulating tumor cells in colorectal cancer

### 2.1 The relationship between MSI-H and CTCs

Colorectal cancers (CRCs) with microsatellite instability-high (MSI-H) are considered to have a better prognosis. The level of MSI-H in CRC is associated with the extent of tumor-infiltrating lymphocytes (TILs). The presence of TILs may partially restrict tumor cell metastasis, potentially by reducing the release of CTCs (23). Microsatellites refer to short tandem repeat sequences scattered throughout the genome (comprising 1–6 or more base pairs, typically repeated 5 to 50 times). When base-pair mismatches or replication errors occur frequently, they are termed microsatellite instability. The accumulation of genetic mutations produces additional tumor antigens, enabling the possibility of immunotherapy. The incidence of MSI-H in CRC is approximately 10–15% (24). However, in clinical practice, the detection rate of MSI-H may fall below 10%, attributed to the high costs and technical complexity of microsatellite testing. This results in many patients missing the opportunity for immunotherapy. Several recent studies have demonstrated the feasibility of identifying immunotherapy-eligible patients more affordably and efficiently by analyzing circulating tumor cells in the blood. Immunotherapy involves the use of immune checkpoint inhibitors (ICIs) to specifically block immune checkpoints such as PD-L1, CTLA-4, and CD47, thereby disrupting the immunosuppressive tumor microenvironment (25).

### 2.2 Perioperative CTC dynamics and MSI status differences

The count of circulating tumor cells (CTCs) correlates with tumor stage, showing statistically significant differences in peripheral blood measurements at various stages and time points (preoperative, intraoperative, postoperative) (26). Notably, the dynamic trends of perioperative CTC counts differed significantly between patients with MSI-H tumors and those with microsatellite−stable (MSS) tumors. The mechanical manipulation–induced tumor cell shedding effect during surgery was pronounced in the MSI-H subtype, with a median CTC count of 37.8, which was significantly higher than the 23.7 observed in the MSS group (P = 0.0328). Postoperative dynamic monitoring revealed a rapid decline in CTC counts from 24 hours to one month after surgery in MSI-H patients, whereas MSS patients exhibited persistently low-level fluctuations or no significant change (23, 27). Overall, perioperative CTC counts were higher in MSI-H patients compared to MSS patients (27).

This finding contradicts intuition, as MSI-H is associated with better survival outcomes, while a CTC count >3 is linked to poorer prognosis. Toh JWT et al. showed the median CTC count for 13 MSS colorectal cancer (CRC) patients at preoperative, intraoperative, and postoperative time points was 1. Conversely, MSI-H CRC patients had median CTC counts exceeding 10 at all measured time points. This paradoxical result was not fully explained in the study. The authors proposed a hypothesis: CTCs shed from MSI-H tumors retain microsatellite instability and carry more immunogenic antigens, potentially eliciting stronger immune responses in peripheral blood and enhancing lymphocyte immunogenicity (23). Studies have indicated that although peripheral CTCs in MSI−H CRC patients are relatively more abundant, their “quality” and functional status may differ from those in MSS patients: CTCs originating from MSI−H tumors harbor indel−induced frameshift mutations that profoundly alter protein amino acid sequences, endowing them with highly immunogenic neoantigens that are readily recognized and cleared by activated T cells, and their survival and metastatic potential may be lower than those of MSS−derived CTCs (28). High PD-L1 expression on the surface of CTCs can bind to PD-1 on T cells, terminating downstream T-cell receptor (TCR) signaling and CD28 co-stimulation, thereby transiently suppressing naïve effector T cells, though its suppressive effect on memory T cells is limited. Consequently, truly metastatic CTC clones may be effectively eliminated by memory T cells (29, 30). Moreover, tumor cell stemness characteristics and inflammatory cytokines (e.g., TNFα, IL-6) promote upregulation of adhesion molecules on tumor cells, facilitating the formation of CTC clusters in peripheral blood (31, 32). Within CTC clusters derived from MSI−H tumors, heterogeneous tumor mutation burdens (TMB) aggregate, leading to clusters containing increased apoptotic markers, which may limit their distant metastatic potential (33).

### 2.3 MSI-dependent prognostic utility of CTCs

This raises important questions: Do CTCs shed from MSI-H and MSS CRC patients have equivalent metastatic potential? Should MSI-H and MSS patients share the same CTC cutoff values? Nearly all clinical studies employ a uniform threshold, namely the FDA-approved CellSearch criterion of ≥3 CTCs per 7.5 mL of blood as an adverse prognostic indicator in metastatic CRC (34). Alsayed et al. proposed that postoperative CTC levels remaining ≥4 cells per 7 mL of blood constitute an independent prognostic factor for overall survival (OS) (27). Toh et al. found that a preoperative median CTC count >10 in MSI-H patients remained associated with favorable prognosis, whereas >3 CTCs in MSS patients indicated adverse outcomes (23). In practice, investigators may stratify CTC counts into categories of 0 vs. ≥1, 3, 4, or 5 (depending on study design), but no studies have specifically calibrated or stratified these cutoffs by MSI subtype. However, Messaritakis et al. developed a molecular assay for CTC detection based on carcinoembryonic antigen-like cellular adhesion molecule 5 (CEACAM5) (35). They found that CEACAM5 mRNA–positive circulating tumor cells (CTCs) were associated with reduced overall survival (11.2 months vs. 19.6 months) and poorer clinical outcomes in patients with MSI−H metastatic CRC (mCRC). Although current evidence for MSI-H CRC is limited, existing studies suggest that CEACAM5-positive CTCs in MSI-H patients may predict poorer clinical outcomes. And post-treatment reduction in CTC burden may be associated with improved prognosis. Therefore, stratifying patients solely on the basis of a CTC count >3 cells/mL—without accounting for MSI status—is inadvisable. In MSI-high colorectal cancer patients, the clinical value of CTC enumeration should be interpreted in conjunction with phenotypic and molecular characteristics of the circulating tumor cells.

Given the limited number and small sample sizes of current studies, the overall evidence remains incomplete. Large-scale, prospective studies are needed to validate whether dynamic changes in CTCs during treatment (including pre- and post-surgery, chemotherapy, or immunotherapy) can reliably predict recurrence risk, progression-free survival, or overall survival in MSI-H CRC patients. Additionally, development of more CTC-related biomarkers is necessary to assess the metastatic and invasive potential of individual shed tumor cells.

Current prognostic models based on CTC counts (e.g., ≥3 per 7.5 mL) do not distinguish MSI status, potentially leading to over-risk stratification of MSI-H patients. In clinical practice, management of MSI-H colorectal cancer patients should incorporate CTC functional characteristics (e.g., PD-L1 expression or CEACAM5 mRNA positivity) and dynamic monitoring of CTC count changes pre- and postoperatively as well as before and after adjuvant therapy to optimize personalized management strategies. Ultimately, integrating these functional CTC assessments with traditional clinicopathological factors will enable more accurate risk stratification, reducing unnecessary interventions in low-risk MSI H individuals and ensuring high-risk patients receive timely escalation of care.

### 2.4 Tumor site and CTC biological characteristics

Tumor location also influences CTC counts, a phenomenon observed in many clinical studies. Left- and right-sided colon cancers differ in tumor characteristics due to disparities in embryological origin, gene expression, and clinical behavior. From an embryological perspective, the demarcation between the left and right colon lies at the distal third of the transverse colon (36). During the fourth week of gestation, the endoderm of the fetus folds and rotates, forming the foregut (blind-ending cranially), hindgut (blind-ending caudally), and midgut, which remains open to the yolk sac via the vitelline duct. The midgut develops into the jejunum, ileum, cecum, ascending colon, and two-thirds of the transverse colon. The hindgut forms the remaining third of the transverse colon, descending colon, and sigmoid colon (37). Anatomically, the right colon is primarily supplied by the superior mesenteric artery, while the left colon is perfused by the inferior mesenteric artery. At the genetic level, right-sided colon cancers often exhibit distinct genetic mutations, higher PD-L1 expression, and elevated microsatellite instability, which may lead to increased infiltration of CD8+ tumor-infiltrating lymphocytes (TILs). Notably, stage II right-sided colon cancers have a higher likelihood of MSI-H (38).

## 3 The role of CTCs in immunotherapy

### 3.1 Expression of PD-L1 in CTCs and mechanisms of immune resistance

Programmed death-ligand 1 (PD-L1), an immunosuppressive protein, is regulated by colorectal tumors (39). PD-L1 expression is fundamentally regulated by the MAPK and PI3K/AKT signaling pathways (40). In addition to the intrinsic regulation by signaling pathways within tumor cells, studies on CTCs have further revealed how tumors promote immune evasion through the expression of immune checkpoints.

Research on CTCs has revealed that oncogenes and tumor suppressor genes facilitate immune evasion by promoting immune checkpoint expression. Previous studies have shown that PD−L1 expression is inversely correlated with KRAS mutations in colorectal cancer, particularly in MSI−H tumors (41). KRAS-mediated repression of interferon regulatory factor 2 (IRF2) results in high expression of CXCL3, which binds to CXCR2 on myeloid-derived suppressor cells (MDSCs) and regulates the immune responses in colorectal cancers (42). KRAS mutations in colorectal cancer are commonly associated with a MSS phenotype and poor response to single−agent immune checkpoint inhibitors; notably, in the KEYNOTE−177 trial, MSI−H CRC patients harboring KRAS or NRAS mutations did not benefit from ICI monotherapy (43). In CTCs with KRAS mutations, the CTLA-4 gene is also highly expressed, with a positive correlation between KRAS and CTLA-4. MDSCs can secrete immunosuppressive factors such as IL−10 and TGF−β to induce Treg expansion, and these CTLA−4–high Tregs further inhibit CD8^+^ T−cell responses (44, 45). Collectively, these findings indicate that KRAS mutations drive immune evasion in colorectal cancer through multifaceted mechanisms, including CXCL3-mediated MDSC recruitment via IRF2 suppression, PD-L1 downregulation in MSI-H tumors, and CTLA-4-dependent Treg expansion, ultimately dampening anti-tumor CD8^+^ T-cell responses and immune escape (46).

### 3.2 Predictive value of PD-L1 expression in CTCs for treatment response

CTCs exhibiting high PD-L1 expression serve as predictive biomarkers, suggesting potential sensitivity to anti-PD-1/PD-L1 monotherapy in these patients (47). Additionally, their presence indicates a persistent immunosuppressive state within the tumor microenvironment (48). Given this dual role, clinicians managing such patients should move beyond monotherapy paradigms. Rational combination strategies—integrating chemotherapy, targeted therapies, or dual immune checkpoint blockade—can synergistically disrupt immune evasion mechanisms, thereby augmenting treatment response and circumventing resistance pathways (49).

The PD-L1-specific inhibitor pembrolizumab has been employed in numerous clinical trials for over 30 cancers, including gastric cancer, colorectal cancer, head and neck cancer, and melanoma (50). In the study by Yue et al., colorectal cancer (CRC) patients undergoing PD-1 blockade therapy with IB1308 were stratified into four groups based on PD-L1 expression levels on circulating tumor cells(CTCs): PD-L1*negative* (MFI<50), PD-L1*low* (50≤MFI<100), PD-L1*medium* (100≤MFI<150), and PD-L1*high* (MFI≥150). This study was the first to propose a PD-L1 expression cutoff value of 20% for CTCs, revealing that patients with PD-L1*high*CTCs (≥20% abundance) achieved a significantly higher objective response rate (ORR: 64% vs. 14%, P<0.001) and prolonged median progression-free survival (4.27 vs. 2.07 months, HR=3.342, P=0.002) compared to those below the threshold. Longitudinal monitoring demonstrated that dynamic reductions in PD-L1*high* CTC counts correlated with therapeutic efficacy (63.6% of disease control patients showed declines, *P*=0.007), whereas stable or elevated PD-L1*high* CTCs predicted progression (84.2% of PD cases) (51). The study conducted by Tan et al. demonstrated that PD-L1 is not only broadly applicable in immunotherapy but also serves as a predictive biomarker. Patients with high baseline PD-L1 expression on circulating tumor cells (CTCs) who received anti-PD-1/PD-L1 monoclonal antibodies combined with conventional chemotherapy regimens showed significantly prolonged progression-free survival (median PFS: 4.9 months vs. 2.2 months, P < 0.0001) (52). The detection method involves isolating CTCs from blood samples using EpCAM antibodies or other surface markers, combined with CD45 for leukocyte exclusion, followed by immunofluorescence staining with PD-L1-specific antibodies (e.g., clones D84TX, 22C3, or KN802) to evaluate PD-L1 expression.

A study evaluated the efficacy of the oral multikinase inhibitor regorafenib based on PD-L1 expression in CTCs obtained from peripheral blood. This study leveraged the advantage of CTCs in reflecting tumor heterogeneity. CTCs were detected in nearly all metastatic colorectal cancer patients (38/40, 95%). Among 17 patients with tumor progression following regorafenib treatment, shorter progression-free survival (PFS) and overall survival (OS) were observed, with PD-L1(+) CTCs present in their blood. These findings suggest that PD-L1-positive tumors may develop resistance to regorafenib. Importantly, such resistance could be detected via CTCs as early as one month after initiating treatment, enabling timely adjustments to therapeutic strategies (53). Regorafenib inhibits the PI3K/AKT/mTOR and RAF/MEK/ERK signaling cascades, thereby inducing immunogenic cell death in tumor cells and promoting the release of ATP, high-mobility group box 1(HMGB1), and other damage‐associated molecular patterns. Concomitantly, these activate T cells to secrete IFN-γ, which, via the JAK–STAT pathway, upregulates PD-L1 expression and engenders an “immune editing” effect that maintains PD-L1 exposure on residual tumor cells, thus furnishing targets for subsequent immunotherapy (54, 55). Consequently, in patients harboring PD-L1–positive CTCs, regorafenib monotherapy demonstrates limited efficacy, whereas its combination with immune checkpoint inhibitors yields significantly enhanced therapeutic responses (56).

### 3.3 CTCs and microsatellite status in multimodal therapy

Circulating tumor cells (CTCs) play a crucial role in monitoring disease progression (PD) and serve as important biomarkers for prognostic assessment and intermediate response evaluation in immunotherapy. CTCs are integral to prognostic stratification in colorectal cancer (CRC) patients, with numerous studies validating their clinical utility. Previously, Bahnassy et al. conducted a prospective cohort study involving 44 CRC patients (Stages I–IV) to evaluate the prognostic significance of changes in CTC counts and microsatellite instability (MSI) profiles before and after curative surgery. The study revealed that a sustained postoperative decline in CTCs, combined with MSI-high (MSI-H) status, served as independent indicators of better progression-free survival (PFS) and overall survival (OS) in advanced-stage patients (P<0.001) (27). This could be attributed to the dynamic evolution of tumor lesions influenced by time, treatment, and surgical intervention, wherein immunotherapy stimulates the expansion of tumor subclones, resulting in changes in the number and molecular characteristics of CTCs (57). In this review, we summarize several studies related to circulating tumor cells (CTCs) in colorectal cancer (CRC). The findings not only underscore the potential value of CTC counts but also provide deeper insights into the feasibility of utilizing CTCs as a liquid biopsy tool (**Table 1**).

**Table 1:** Summarization of clinical trials related to the CTCs in Colorectal immunotherapy.

Number of CRC patients	Stage	Rate of MSI-H/PD-L1(+)	Results	Reference
5	Advanced tumor	0.6(3/5)	The dynamic changes of CTC could indicate the therapeutic response at early time	(51)
8	Advanced tumor	0.75(6/8)	The clinical benefit of anti-PD-1/PD-L1 monotherapy in patients with advanced colorectal cancer is limited, which may be related to the low proportion of MSI-H.	(52)
100	I-IV	0.44(44/100)	In 100 patients, MSI and CTC serve as accurate, reliable, and sensitive diagnostic and prognostic biomarkers for survival and outcome in CRC patients	(27)
17	I-IV	0.24(4/17)	In MSI-H CRC, CTC release was increased before, during and after operation. The number of CTC in peripheral blood of MSI-H tumor patients was much higher than that of MSS patients. When using CTC as a prognostic predictor, the same cutoff value should not be used for both	(23)
198	I-IV	0.05(8/163)	In 179 patients, both MSI-H and CTC elevation were associated with decreased PFS and OS, but there was no significant difference, and CEACAM5mRNA positive CTCS were considered to be predictors of poor prognosis (decreased OS) in patients with metastatic CRC	(35)
31	I, III, IV	0.06(2/31)	A comprehensive genomic and transcriptomic analysis of colorectal source CTCS revealed significant heterogeneity among CTCS, which may explain the relatively small number of metastases compared to the number of CTCS present in the bloodstream (42), and suggests that only a small percentage of CTCS are actually able to form new lesions	(58)
62	I-IV	0.16(10/52)	The presence of more CTCs was significantly associated with advanced cancer stage (p = 0.045) and adenocarcinoma subtype	(46)

## 4 Mechanisms of immune evasion by circulating tumor cells in the tumor microenvironment

### 4.1 Macrophage-CTC interaction

Circulating tumor cells (CTCs), as critical mediators of primary tumors and distant metastases, play a pivotal role in cancer initiation, progression, and metastasis through interactions with the tumor microenvironment (TME) (**Figure 1**) (59). The TME in colorectal cancer comprises stromal and immune cells that regulate immune suppression and inflammation (60). Tumor-associated macrophages (TAMs), the predominant immune cells in the TME, exhibit functional polarization toward either the M1 or M2 phenotype, modulated by tumor and stromal signals. Advances in gene expression profiling, morphology, and single-cell RNA sequencing have provided deeper insights into TAM heterogeneity (61). In the TME, M1 macrophage polarization is induced by recognizing pathogen-associated molecular patterns (e.g., lipopolysaccharide [LPS]) and type 1 helper T cell (Th1) cytokines, such as interferon-γ (IFN-γ) and tumor necrosis factor-α (TNF-α). M1 macrophages primarily function in innate immunity to combat infections and tumors. In contrast, M2 polarization is driven by interleukin-4 (IL-4), interleukin-13 (IL-13), macrophage colony-stimulating factor (M-CSF), and transforming growth factor-β (TGF-β), contributing to pro-tumor characteristics (62, 63). Most TAMs exhibit the M2 phenotype, significantly promoting tumor cell survival, proliferation, and immune evasion by enhancing immunosuppression. This ultimately leads to cancer progression, chemoresistance, and metastasis. Within the TME, signals such as IL-10, CCL2, CSF-1, VEGF, and IL-6 secreted by cancer cells, adaptive immune cells, fibroblasts, and TAMs recruit and differentiate monocytes into M2-like TAMs (64, 65).

*In vitro* and *in vivo* studies suggest that CTCs may originate from the fusion of tumor cells with hematopoietic or myeloid cells, particularly macrophages (66). This intercellular interaction relies on the formation of membrane protrusions, which facilitate signal exchange over short distances (tens of micrometers) and long distances (hundreds of micrometers) through direct cell contact. Among these structures, the most extensively studied are protrusions derived from filopodia, including cytochromes and tunneling nanotubes (TNTs). TNTs represent the extended filopodia, connecting previously non-adjacent cells through a process referred to as “protrusion elongation” (67). M2−polarized macrophages more readily form membrane protrusions and fuse with tumor cells via tunneling nanotubes than M1 macrophages. Wei et al. showed that M2−derived IL−6 activates the JAK2/STAT3 pathway in tumor cells, leading to STAT3 nuclear translocation and repression of miR−506−3p, which upregulates FoxQ1. FoxQ1 induces epithelial–mesenchymal transition (EMT), yielding CTCs with enhanced invasiveness, and drives C-C motif chemokine ligand 2 (CCL2) secretion to recruit more M2 macrophages, creating a pro−metastatic feedback loop (68).

Recent studies have revealed that TNT-mediated paracrine signaling and juxtacrine interactions between tumor cells and macrophages play crucial roles in shared molecular pathways that enhance cell migration and invasion. The interaction between macrophages and tumor cells in the TME predominantly depends on classical paracrine mechanisms (69). Specifically, macrophages secrete epidermal growth factor (EGF), which binds to epidermal growth factor receptors (EGFR) on tumor cells, activating downstream signaling pathways and inducing colony-stimulating factor 1 (CSF-1) secretion. CSF-1 binds to its receptor to recruit macrophages to tumor sites. This paracrine loop, mediated by tumor cell-secreted CSF-1 and macrophage-secreted EGF, is critical in facilitating the co-migration of tumor cells and macrophages toward blood vessels. Once tumor cells enter the bloodstream, they can be detected as circulating hybrid cells or CTCs (70).

### 4.2 Neutrophils-CTC interaction

Surgery-induced dissemination of circulating tumor cells (CTCs) and the accompanying inflammatory response promote the growth and metastasis of occult tumors by constructing a supportive tumor microenvironment (TME) (71). Neutrophil extracellular traps(NETs), composed of depolymerized DNA fibers modified by proteins secreted by activated neutrophils, capture CTCs in cases of severe postoperative sepsis. This fosters early adhesion of tumor cells to distant organs, creating favorable conditions for tumor metastasis (72). These CTC-neutrophil clusters form a “shield” around tumor cells, not only physically isolating CTCs but also suppressing NK cell-mediated cytotoxicity. Additionally, they promote tumor cell evasion by releasing cytokines such as IL-1B, MMP-9, and HMGB1 (62, 73).

The significance of NETs-related genes in immunotherapy and cancer treatment has garnered increasing attention. Studies have demonstrated that the expression levels of NETs-related genes (e.g., H3Cit, NE, MPO) are closely associated with an immunosuppressive microenvironment and the response to cancer immunotherapy (74). During immune checkpoint inhibitor therapy, changes in the expression of these genes can serve as indicators of immune response intensity. Transcriptome analyses have identified 19 genes associated with NETs formation, leading to the establishment of a NETs scoring system. This score has been found to negatively correlate with cancer patient prognosis (75, 76). Furthermore, the expression levels of NETs-related genes across different tumor types are intricately linked to mechanisms of tumor immune evasion, immunosuppression, and cancer progression. In CTC-related studies, the interaction between NETs and CTCs promote tumor invasion, metastasis, and immune evasion through multiple mechanisms. First, NETs physically entrap CTCs, enhancing their intravascular retention and adhesion while shielding them from immune clearance. Concurrently, NETs release proteolytic enzymes—such as matrix metalloproteinase-9 (MMP-9) and neutrophil elastase—that degrade the extracellular matrix, thereby creating a permissive niche for tumor cell invasion and migration. They also secrete cytokines like interleukin-8 (IL-8) and transforming growth factor-β (TGF-β), which induce EMT in CTCs and further augment their migratory and invasive capabilities. Moreover, NET-associated high-mobility group box 1 (HMGB1) activates the RAGE and TLR4 signaling pathways to drive the release of pro-inflammatory mediators (e.g., CXCL2, IL-8), recruiting additional neutrophils and establishing a pro-metastatic inflammatory microenvironment. In the realm of immune regulation, NETs discharge immunosuppressive factors—including programmed death ligand-1 (PD-L1), reactive oxygen species (ROS), and arginase-1 (ARG1)—to inhibit T cell and natural killer (NK) cell cytotoxicity, while fostering the accumulation of myeloid-derived suppressor cells (MDSCs) and regulatory T cells (Tregs), thereby remodeling the tumor microenvironment into an immunosuppressive state (77, 78).

### 4.3 Other immune cells

Circulating tumor cells (CTCs) evade immune surveillance through multiple mechanisms, facilitating tumor metastasis and modulating the function of immune cells. First, CTCs escape NK cell surveillance by upregulating HLA-I through the cGAS-STING-IFNβ-HLA signaling pathway (79). Additionally, CTCs enhance immune evasion by upregulating N-cadherin, a natural ligand of the NK cell receptor KLRG1. The interaction between N-cadherin and KLRG1 inhibits NK cell cytotoxicity, induces NK cell differentiation, and increases the proportion of KLRG1+ cells, leading to NK cell exhaustion and impaired antitumor efficacy (80). Furthermore, CTCs achieve immune evasion through interactions with T cells, particularly via exosome-derived CD45 transferred to the tumor cell surface, forming CD45+ CTCs. These CD45+ CTCs inhibit TCR signaling through CD45-CD45 interactions with T cells, reducing T-cell cytotoxic responses and accelerating tumor metastasis (81). These findings elucidate the complex interaction mechanisms between CTCs and immune cells, providing new insights into tumor immune evasion and highlighting potential therapeutic targets for CTCs-targeted immunotherapy. The mechanisms of CTC immune evasion involving various immune cells are summarized in **Table 2**.

**Table 2:** the escape mechanism of CTC in immune microenvironment.

Immune cell	Mediator	Forming pathway	Escape mechanism	Reference
Macrophages	M1 polarization	Induced by pathogen-associated molecular patterns (PAMPs; e.g., LPS) and Th1 cytokines (e.g., IFN-γ, TNF-α). Induced by pathogen-associated molecular patterns (PAMPs; e.g., LPS) and Th1 cytokines (e.g., IFN-γ, TNF-α).	M1 macrophages contribute to anti-tumor immunity by releasing pro-inflammatory cytokines and activating cytotoxic T cells.	(70)
	M2 polarization	Driven by IL-4, IL-13, M-CSF, and TGF-β.	M2 macrophages secrete immunosuppressive factors (IL-10, CCL2, MMP9), recruit regulatory T cells (Tregs), and upregulate PD-L1 to inhibit T-cell responses.	(70)
Neutrophils	NETs (Neutrophil Extracellular Traps)	Activated by pathogens or inflammatory signals via NETosis (chromatin release with proteases like NE, MPO).	NETs physically trap CTCs, shield them from immune clearance, degrade extracellular matrix (via MMP-9), and recruit immunosuppressive cells (MDSCs, Tregs).	(82)
NK cell	mesenchymal stromal cells (MSCs)	CTCs secrete cGAMP to activate the STING pathway in MSCs.	STING-IFNβ signaling upregulates HLA-I on CTCs, enabling evasion from NK cell surveillance.	(80)
T cell	CD45+ CTCs	Transfer of CD45 from exosomes to CTC surfaces.	CD45+ CTCs inhibit TCR signaling via CD45-CD45 interactions, reducing T-cell cytotoxicity and promoting immune evasion.	(81)

## 5 Innovation and progress in circulating tumor cell screening technologies

### 5.1 Screening and application of novel genetic markers

Liquid biopsy has emerged as a transformative approach in oncology, encompassing diverse biomarkers such as circulating tumor DNA (ctDNA), microRNAs (miRNAs), extracellular vesicles (EVs), and CTCs to guide precision medicine (83). These components collectively provide a non-invasive window into tumor dynamics, enabling real-time monitoring of disease progression and therapeutic response. Among these, CTCs hold unique value as intact cellular entities that reflect both genetic and functional characteristics of tumors, offering insights into metastasis and immune evasion mechanisms.

In CTC screening, common genetic mutations associated with colorectal cancer include KRAS and BRAF mutations. KRAS mutations lead to the activation of KRAS protein, which promotes tumor cell proliferation. BRAF gene mutations, in the B-Raf proto-oncogene serine/threonine kinase, are closely related to metastasis and drug resistance. However, the mutation rates of KRAS and BRAF in colorectal cancer patients are only 40% and 10%, respectively (84–86). Advances in proteomic technologies, such as those highlighted in hepatocellular carcinoma (HCC) biomarker research, could enhance CTC characterization in CRC (87). For example, mass spectrometry and pathway analysis—tools pivotal for uncovering PI3K/AKT/mTOR dysregulation in HCC—could likewise characterize post-translational modifications and protein interactions in CTCs, thereby refining prognostic assessments and therapeutic strategies in CRC. Notably, CTC detection shows a 77% concordance with tumor tissue profiling (88). The application of DNA microarray technology not only allows for the detection of mutated genes that are masked by wild-type DNA in contaminating leukocytes, but it also offers lower costs and higher sensitivity. With the help of DNA microarrays, research can progress to the coding level. Commonly analyzed mutations include KRAS mutations in exon 2 (codons 12 and 13), 3 (codon 61), and 4 (codon 146), NRAS mutations in exon 2 (codons 12 and 13), and BRAF mutations in exon 15 (V600E) (89).

Cluster differentiation (CD) markers are emerging as potential targets. The expression of CD45 in CTCs is consistent with that in corresponding tumor tissues, while CD47 expression is significantly upregulated and closely associated with immune evasion by cancer cells (90, 91). Mass spectrometry has been used to localize protein glycosylation, revealing that cancer-associated glycans such as Sialyl-Tn (STn) are expressed in most advanced gastrointestinal cancers, including colorectal cancer, but are minimally expressed or absent in normal tissues. Glycans, by modulating the activity of cell-surface glycosyltransferases in tumor cells, induce aberrant glycosylation and the emergence of truncated glycan epitopes (e.g., Tn and Sialyl-Tn). These truncated glycan structures contribute to enhanced metastatic potential, invasive capacity, and immune evasion (92, 93). In CTCs, STn expression is considered one of the primary drivers of metastasis and a significant downstream biological target. Studies have shown that STn(+) CTCs can also be captured, with an incidence three times higher than that of EpCAM(+) CTCs (94).

### 5.2 Breakthroughs in emerging screening technologies: microfluidics and molecular aptamers

#### 5.2.1 CellSearch system

Counting circulating tumor cells (CTCs) is technically challenging, as CTCs constitute less than 0.004% of all mononuclear cells (95). The CellSearch System (Veridex) is the most widely utilized antibody-based isolation technology and the only method approved by the U.S. Food and Drug Administration (FDA) for detecting CTCs in the blood of patients with certain cancers (96). The CellSearch System enriches tumor cells using ferromagnetic beads coated with EpCAM antibodies. EpCAM stands for Epithelial Cell Adhesion Molecule. Colorectal cancer arises from the epithelial cells lining the colon or rectum, and these tumor cells are highly likely to express EpCAM on their surface (97). Consequently, EpCAM offers high specificity, as colorectal carcinoma cells generally retain epithelial characteristics even during metastasis. In contrast, CD45 serves as a pan−leukocyte marker for negative selection, effectively labeling and depleting the vast majority of immune cells to minimize contamination (98). These magnetic beads specifically bind to EpCAM-positive CTCs, capturing them and isolating them from the patient’s peripheral blood (99). Flow cytometry is subsequently used to further sort out and remove leukocytes, ultimately isolating individual CTCs (100). CellSearch is currently the most established CTC‐enrichment method, and the genomic mutational profile of isolated CTCs is largely concordant with that of the primary tumor. However, its low sensitivity has constrained the informational value of CTC enumeration in this disease (101).

#### 5.2.2 Microfluidics-based technology

Microfluidics-based cell sorting methods leverage fluid dynamics and external forces (such as magnetic fields, electric fields, acoustic waves, and optical forces) to separate cells based on their physical and biological properties (102). Microfluidic technology can organize CTCs into monolayers in a few minutes (103). Yang et al. developed a label-free wedge-shaped microfluidic chip called CTC-Δchip. This enrichment technique relies on size-based filtration, using nano- to micron-scale pores to isolate CTCs, which are larger and stiffer in shape, from blood cells (104). The self-assembled cell array (SACA) has been demonstrated as a reliable platform for CTC enumeration. When combined with a 3D-microDialysis chip, SACA enables image analysis to be completed in under 4 hours and exhibits high sensitivity in detecting one CTC among 105^55 cells. Additionally, SACA combined with carcinoembryonic antigen (CEA) serves as a powerful risk stratification tool. Patients with preoperative CTC counts >4 and CEA levels >5 ng/mL had poorer progression-free survival (PFS) compared to others (105). Microfluidic technology, as a biophysical isolation method, exploits the intrinsic physical properties of CTCs and blood cells. It enables the capture of EpCAM‐negative CTCs, which often exhibit greater invasiveness and metastatic potential, while bypassing the labor‐intensive, multi‐step analyses required by biochemical isolation and thereby significantly shortening enrichment time—making it well suited for real‐time intraoperative monitoring (106). However, megakaryocytes or activated leukocytes of similar size may be misclassified as CTCs. Issues related to false positives (specificity) and false negatives (sensitivity) remain major challenges for immunomagnetic detection technologies in CTC isolation (107).

#### 5.2.3 Application of molecular aptamers in CTC detection

W3 is an aptamer that has long been regarded as a predictive factor for colorectal cancer (CRC) metastasis. A molecular beacon based on W3 (MAB-W3-3G) can act as a molecular probe, specifically capturing CTCs in the bloodstream (108). The molecular aptamer beacon combines the advantages of both aptamers and molecular beacons, offering not only the specificity of aptamers in detecting targets but also the convenience of molecular beacons in operationally detecting those targets (109). Lu et al. (2023) used SELEX technology to obtain the aptamer W3 from CRC cells and employed W3 as a specific recognition probe for the molecular beacon (MAB). In the stem region of MAB, some base sequences were modified to maintain stability, and the shortest sequence out of four variants was selected, named W3-3. This was further used to construct a monoclonal antibody, MAB-W3-3G. In a real blood sample validation using 14 healthy blood donors and 28 CRC patients, no positive cells were detected in the blood samples from healthy donors. In 28 CRC patient samples, 75% of the patients tested positive for CTCs (21/28), indicating that MAB-W3-3G-based imaging can specifically detect cancer cells in whole blood. Notably, the number of CTCs in metastatic patients was significantly higher than in non-metastatic patients (6.4 ± 2.0, n = 8 vs 2.3 ± 0.5, n = 20) (110). The W3 aptamer can be conjugated to either quantum-dot probes or molecular beacons (MAB-W3-3G) to enable one-step capture and quantification of metastatic CTCs in patient blood, and it also selectively recognizes EphA2‐bearing exosomes, achieving a detection sensitivity of 8.4×10^6^ particles/ml. Moreover, W3 exhibits excellent stability in both plasma and culture medium—retaining full activity over a 3-hour period—and is compatible with live‐cell imaging and microplate‐based fluorescence assays, offering operational simplicity and reduced sample loss (111). However, as it targets only a single EphA2 marker, it is prone to interference from tumor cell expression heterogeneity and nonspecific adsorption in whole blood (108). Additionally, MAB-W3-3G shows elevated background signals at 37 °C, indicating that further probe optimization is required. Its performance has thus far been validated only in a small cohort, underscoring the need for larger, multicenter clinical trials to assess diagnostic concordance and prognostic value (110).

#### 5.2.4 Applications of nanomaterials in biomedicine

Nanomaterials are widely used in the biomedical field due to their unique physicochemical properties, such as high surface area and good biocompatibility (112). Common nanomaterials include gold, magnetic, and silica-based materials, which typically exist in the form of nanoparticles, nanostructures, or nanowires (113). Gold nanomaterials, owing to their excellent conductivity, stability, and increased surface area, are able to effectively interact with various biomolecules. They are commonly used in molecular detection and imaging applications, such as fluorescence imaging and Raman spectroscopy. Furthermore, the high biocompatibility of gold nanoparticles allows them to enter the body and be used for the separation and detection of CTCs (circulating tumor cells) and CCSCs (cancer stem cells) (114). In related studies, gold nanorods, when combined with targeted antibodies like EpCAM, CD44, etc., utilize surface-enhanced Raman scattering (SERS) technology to achieve highly sensitive and multiplexed detection of CTCs from blood samples (115). Silica-based nanomaterials are widely used in CTC detection platforms due to their excellent chemical stability and biocompatibility (116). The nanoparticles or nanostructures of silica materials enhance interactions with cells, improving the efficiency of CTC capture. They can also be integrated with optical detection technologies and microfluidic devices to provide higher sensitivity and specificity (117).

Despite the development of various commercial CTC detection systems, which have made progress in laboratory settings, these methods have not been widely adopted in clinical practice due to certain limitations. Future clinical studies should address how to improve CTC capture efficiency, simplify identification methods, reduce cell loss, and optimize the clinical applicability of nanotechnology to enhance the practical value of CTC detection in early cancer diagnosis, monitoring, and therapy(**Table 3**) (118).

**Table 3:** comparison of CTC detection technologies.

Method	Advantages	Disadvantages	Reference
CellSearch	1.FDA-approved, standardized method for CTC enumeration. 2.High specificity via EpCAM-based immunomagnetic capture. 3.Validated prognostic utility in multiple cancers.	1.Low sensitivity for EpCAM-negative CTCs. 2.Limited ability to capture mesenchymal or hybrid CTCs. 3.High cost and technical complexity.	(99–101)
Microfluidic	1.Label-free isolation based on physical properties (size, deformability). 2.Captures EpCAM-negative CTCs. 3.Rapid processing, suitable for intraoperative monitoring.	Risk of false positives due to leukocyte contamination.	(102–107)
Molecular Aptamer	1.High specificity and affinity for target biomarkers (e.g., EphA2). 2.Simple operation and reduced sample loss	1.Targeting a single marker (e.g., EphA2) is susceptible to heterogeneity and non−specific adsorption. 2.Elevated background signal at physiological temperature (37 °C) requires probe optimization-. 3.Validation limited to small cohorts; lacks large multicenter data	(108–111)
Nanomaterials	1.High surface area enhances capture efficiency. 2.Multifunctional integration (e.g., SERS, fluorescence). 3.Customizable surface modifications for targeted capture.	1.Potential cytotoxicity and biocompatibility concerns. 2.Challenges in complex synthesis and standardization. 3.Signal interference within whole blood environments.	(114, 115, 118)

## 6 Current status and challenges

Liquid biopsy, particularly the detection of circulating tumor cells (CTCs), has shown tremendous potential in the immunotherapy of colorectal cancer (CRC). However, challenges remain in improving the sensitivity and specificity of screening due to the short half-life of CTCs in circulation and the significantly higher concentrations of CTCs in the portal vein/mesenteric vein blood compared to central venous blood (119, 120). Furthermore, the tumor heterogeneity of CRC further complicates CTC detection (121). CRC exhibits significant intra-tumoral and inter-tumoral heterogeneity, with phenotypic and genotypic differences between metastatic and primary lesions. This makes CTC capture and analysis more complex. Such heterogeneity not only affects the efficiency of CTC detection but also limits their utility as prognostic and predictive biomarkers (122). For example, the metastatic routes and hemodynamic changes in tumors may lead to different biological characteristics of CTCs, increasing the technical difficulty of detecting them (123). Additionally, current methods for CTC isolation and characterization are still immature, and there is a lack of standardized operating procedures (SOPs). Differences in reagents, equipment, and operational procedures used in different laboratories make the reproducibility and comparability of research results difficult. Therefore, developing standardized operating procedures (SOPs) and validation methods is crucial for the widespread application of liquid biopsy technologies (124).

To overcome these challenges, scientists are developing liquid biopsy technologies with higher sensitivity and specificity. One of the current focuses of research is the improvement of CTC separation techniques and the use of multi-biomarker combined analysis. Traditional CTC capture methods typically rely on surface markers, such as epithelial cell adhesion molecule (EpCAM). However, these methods are prone to selective bias. As a result, researchers are exploring multi-molecular marker-based combined analyses to enhance the comprehensiveness and accuracy of CTC capture (125–127). Multi-marker strategies not only improve CTC capture efficiency but also address the limitations of relying on a single marker, which may miss specific types of CTCs (128). Yu et al. noted that the integration of genomics, transcriptomics, proteomics, and metabolomics enables the construction of comprehensive molecular profiles for early tumor detection and therapeutic intervention. These approaches—particularly when coupled with artificial intelligence (AI) and machine learning (ML)–driven data analysis—enhance biomarker discovery by detecting subtle patterns within heterogeneous datasets, thereby facilitating real-time monitoring of treatment response and disease progression (129). The integration of AI and ML technologies provides new insights into the analysis of liquid biopsy data. These technologies are capable of efficiently processing complex multidimensional data, enabling researchers to extract valuable clinical information from liquid biopsies (130, 131). For example, AI algorithms can be used to analyze the morphological features of CTCs, their gene expression profiles, and their relationship with tumor progression, thereby improving the accuracy of liquid biopsies in tumor diagnosis, prognosis evaluation, and monitoring treatment responses (132).

In addition to technical advancements, conducting multicenter, large-scale clinical studies and fostering multidisciplinary collaboration are essential for transitioning liquid biopsy technologies from research to clinical practice (133, 134). Currently, the clinical application of liquid biopsy in colorectal cancer is still in its early stages. Although some clinical studies have shown that liquid biopsy has potential for early screening, treatment response prediction, and prognosis evaluation of CRC, its diagnostic accuracy and sensitivity still face many challenges (135). For example, liquid biopsy may struggle with detecting mutations with low allele frequencies, such as microsatellite instability (MSI), due to insufficient sensitivity when mutation frequencies are too low, especially in early-stage tumors or localized cancers (low tumor mutational burden, TMB). Additionally, current liquid biopsy technologies, particularly CTC detection based on next-generation sequencing (NGS), are limited by low signal-to-noise ratios and sample contamination (e.g., non-tumor cells such as immune and hematopoietic cells in the blood) (136–138). Furthermore, combining liquid biopsy with other components, such as circulating tumor DNA (ctDNA), may significantly enhance the sensitivity and specificity of early colorectal cancer detection (139). This approach could further help identify advanced colorectal cancer patients who are suitable for immunotherapy or surgical resection (140).

## 7 Conclusion and future prospects

This article summarizes the clinical significance of circulating tumor cells (CTC) in the immune treatment of colorectal cancer (CRC), particularly in the roles of prognosis assessment, immune escape, drug resistance mechanisms, and tumor microenvironment (TME) interactions. Research shows that CTCs have significant potential in treatment monitoring and immunotherapy in CRC patients. MSI-H patients often exhibit higher CTC counts, which may suggest better prognoses in response to immunotherapy. Additionally, the role of PD-L1 positive CTCs in immune resistance further validates the potential of CTCs as predictors of drug response.

Looking forward, the use of CTCs as a liquid biopsy tool for CRC, particularly in personalized treatment and early screening, still holds vast developmental potential. With the discovery of new genetic markers and advancements in screening technologies, CTCs could provide more precise prognosis predictions and treatment response evaluations for CRC patients.

## References

[1] Sung JJY Chiu HM Lieberman D Kuipers EJ Rutter MD Macrae F. Third asia-pacific consensus recommendations on colorectal cancer screening and postpolypectomy surveillance. Gut. (2022) 71:2152–66. doi: 10.1136/gutjnl-2022-327377 36002247

[2] Bretthauer M Loberg M Wieszczy P Kalager M Emilsson L Garborg K. Effect of colonoscopy screening on risks of colorectal cancer and related death. N Engl J Med. (2022) 387:1547–56. doi: 10.1056/NEJMoa2208375 36214590

[3] Lin JS Perdue LA Henrikson NB Bean SI Blasi PR. Screening for colorectal cancer: updated evidence report and systematic review for the us preventive services task force. JAMA. (2021) 325:1978–98. doi: 10.1001/jama.2021.4417 PMC13509038 34003220

[4] Carney BW Gholami S Fananapazir G Sekhon S Lamba R Loehfelm TW. Utility of combined gadoxetic acid and ferumoxytol-enhanced liver mri for preoperative detection of colorectal cancer liver metastases: A pilot study. Acta Radiol. (2023) 64:1357–62. doi: 10.1177/02841851221136499 36437569

[5] Adam R de Gramont A Figueras J Kokudo N Kunstlinger F Loyer E. Managing synchronous liver metastases from colorectal cancer: A multidisciplinary international consensus. Cancer Treat Rev. (2015) 41:729–41. doi: 10.1016/j.ctrv.2015.06.006 26417845

[6] Gvozdenovic A Aceto N. Emp1-positive cells found guilty of metastatic relapse in colorectal cancer. Dev Cell. (2022) 57:2673–4. doi: 10.1016/j.devcel.2022.11.017 36538891

[7] Tsai KY Huang PS Chu PY Nguyen TNA Hung HY Hsieh CH. Current applications and future directions of circulating tumor cells in colorectal cancer recurrence. Cancers (Basel). (2024) 16(13):2316. doi: 10.3390/cancers16132316 39001379 PMC11240518

[8] Thibaudin M Fumet JD Chibaudel B Bennouna J Borg C Martin-Babau J. First-line durvalumab and tremelimumab with chemotherapy in ras-mutated metastatic colorectal cancer: A phase 1b/2 trial. Nat Med. (2023) 29:2087–98. doi: 10.1038/s41591-023-02497-z PMC10427431 37563240

[9] Patel SP Alonso-Gordoa T Banerjee S Wang D Naidoo J Standifer NE. Phase 1/2 study of monalizumab plus durvalumab in patients with advanced solid tumors. J Immunother Cancer. (2024) 12(2):e007340. doi: 10.1136/jitc-2023-007340 38309722 PMC10840023

[10] Kolencik D Narayan S Thiele JA McKinley D Gerdtsson AS Welter L. Circulating tumor cell kinetics and morphology from the liquid biopsy predict disease progression in patients with metastatic colorectal cancer following resection. Cancers (Basel). (2022) 14(3):642. doi: 10.3390/cancers14030642 35158910 PMC8833610

[11] Yang X Zhang Z Bi X. A nomogram for predicting colorectal cancer liver metastasis using circulating tumor cells from the first drainage vein. Eur J Surg Oncol. (2024) 50:108579. doi: 10.1016/j.ejso.2024.108579 39121633

[12] Zhang W Xu F Yao J Mao C Zhu M Qian M. Single-cell metabolic fingerprints discover a cluster of circulating tumor cells with distinct metastatic potential. Nat Commun. (2023) 14:2485. doi: 10.1038/s41467-023-38009-3 37120634 PMC10148826

[13] Galvis MM Romero CS Bueno TO Teng Y. Toward a new era for the management of circulating tumor cells. Adv Exp Med Biol. (2021) 1286:125–34. doi: 10.1007/978-3-030-55035-6_9 PMC8647934 33725350

[14] Giuliano AE Connolly JL Edge SB Mittendorf EA Rugo HS Solin LJ. Breast cancer-major changes in the american joint committee on cancer eighth edition cancer staging manual. CA Cancer J Clin. (2017) 67:290–303. doi: 10.3322/caac.21393 28294295

[15] Nabors LB Portnow J Ahluwalia M Baehring J Brem H Brem S. Central nervous system cancers, version 3.2020, nccn clinical practice guidelines in oncology. J Natl Compr Canc Netw. (2020) 18:1537–70. doi: 10.6004/jnccn.2020.0052 33152694

[16] Schaeffer EM Srinivas S Adra N An Y Barocas D Bitting R. Prostate cancer, version 4.2023, nccn clinical practice guidelines in oncology. J Natl Compr Canc Netw. (2023) 21:1067–96. doi: 10.6004/jnccn.2023.0050 37856213

[17] Gradishar WJ Moran MS Abraham J Aft R Agnese D Allison KH. Breast cancer, version 3.2022, nccn clinical practice guidelines in oncology. J Natl Compr Canc Netw. (2022) 20:691–722. doi: 10.6004/jnccn.2022.0030 35714673

[18] Manso T Kushwaha A Abdollahi N Duroux P Giudicelli V Kossida S. Mechanisms of action of monoclonal antibodies in oncology integrated in imgt/mab-db. Front Immunol. (2023) 14:1129323. doi: 10.3389/fimmu.2023.1129323 37215135 PMC10196129

[19] Sharma P Siddiqui BA Anandhan S Yadav SS Subudhi SK Gao J. The next decade of immune checkpoint therapy. Cancer Discov. (2021) 11:838–57. doi: 10.1158/2159-8290.CD-20-1680 33811120

[20] Thomas J Leal A Overman MJ. Clinical development of immunotherapy for deficient mismatch repair colorectal cancer. Clin Colorectal Cancer. (2020) 19:73–81. doi: 10.1016/j.clcc.2020.02.002 32173280

[21] Wang DK Zuo Q He QY Li B. Targeted immunotherapies in gastrointestinal cancer: from molecular mechanisms to implications. Front Immunol. (2021) 12:705999. doi: 10.3389/fimmu.2021.705999 34447376 PMC8383067

[22] Mei WJ Mi M Qian J Xiao N Yuan Y Ding PR. Clinicopathological characteristics of high microsatellite instability/mismatch repair-deficient colorectal cancer: A narrative review. Front Immunol. (2022) 13:1019582. doi: 10.3389/fimmu.2022.1019582 36618386 PMC9822542

[23] Toh JWT Lim SH MacKenzie S de Souza P Bokey L Chapuis P. Association between microsatellite instability status and peri-operative release of circulating tumour cells in colorectal cancer. Cells. (2020) 9(2):425. doi: 10.3390/cells9020425 32059485 PMC7072224

[24] Ierano C Righelli D D’Alterio C Napolitano M Portella L Rea G. In pd-1+ Human colon cancer cells nivolumab promotes survival and could protect tumor cells from conventional therapies. J Immunother Cancer. (2022) 10(3):e004032. doi: 10.1136/jitc-2021-004032 35246475 PMC8900051

[25] Dompe C Chojnowska A Ramlau R Nowicki M Alix-Panabieres C Budna-Tukan J. Unveiling the dynamics of circulating tumor cells in colorectal cancer: from biology to clinical applications. Front Cell Dev Biol. (2024) 12:1498032. doi: 10.3389/fcell.2024.1498032 39539964 PMC11557528

[26] Hardingham JE Grover P Winter M Hewett PJ Price TJ Thierry B. Detection and clinical significance of circulating tumor cells in colorectal cancer–20 years of progress. Mol Med. (2015) 21 Suppl 1:S25–31. doi: 10.2119/molmed.2015.00149 PMC4661051 26605644

[27] Alsayed A Salem SE El Serafi MM Abdellateif MS Zekri AN Mohanad M. Assessment of the circulating tumor cells and microsatellite instability in colorectal cancer patients: prognostic and diagnostic value. Onco Targets Ther. (2021) 14:1937–51. doi: 10.2147/OTT.S292551 PMC7981167 33758513

[28] Ambrosini M Manca P Nasca V Sciortino C Ghelardi F Seligmann JF. Epidemiology, pathogenesis, biology and evolving management of msi-H/dmmr cancers. Nat Rev Clin Oncol. (2025) 22(6):385-407. doi: 10.1038/s41571-025-01015-z 40181086

[29] Lin X Kang K Chen P Zeng Z Li G Xiong W. Regulatory mechanisms of pd-1/pd-L1 in cancers. Mol Cancer. (2024) 23:108. doi: 10.1186/s12943-024-02023-w 38762484 PMC11102195

[30] Gu X Wei S Lv X. Circulating tumor cells: from new biological insights to clinical practice. Signal Transduct Target Ther. (2024) 9:226. doi: 10.1038/s41392-024-01938-6 39218931 PMC11366768

[31] Fumagalli A Oost KC Kester L Morgner J Bornes L Bruens L. Plasticity of lgr5-negative cancer cells drives metastasis in colorectal cancer. Cell Stem Cell. (2020) 26:569–78 e7. doi: 10.1016/j.stem.2020.02.008 32169167 PMC7118369

[32] Schuster E Taftaf R Reduzzi C Albert MK Romero-Calvo I Liu H. Better together: circulating tumor cell clustering in metastatic cancer. Trends Cancer. (2021) 7:1020–32. doi: 10.1016/j.trecan.2021.07.001 PMC8541931 34481763

[33] Li Q Geng S Luo H Wang W Mo YQ Luo Q. Signaling pathways involved in colorectal cancer: pathogenesis and targeted therapy. Signal Transduct Target Ther. (2024) 9:266. doi: 10.1038/s41392-024-01953-7 39370455 PMC11456611

[34] Magri V Marino L Nicolazzo C Gradilone A De Renzi G De Meo M. Prognostic role of circulating tumor cell trajectories in metastatic colorectal cancer. Cells. (2023) 12(8):1172. doi: 10.3390/cells12081172 37190081 PMC10136568

[35] Messaritakis I Sfakianaki M Vogiatzoglou K Koulouridi A Koutoulaki C Mavroudis D. Evaluation of the role of circulating tumor cells and microsatellite instability status in predicting outcome of advanced crc patients. J Pers Med. (2020) 10(4):235. doi: 10.3390/jpm10040235 33217974 PMC7712177

[36] Bergen ES Scherleitner P Ferreira P Kiesel B Muller C Widhalm G. Primary tumor side is associated with prognosis of colorectal cancer patients with brain metastases. ESMO Open. (2021) 6:100168. doi: 10.1016/j.esmoop.2021.100168 34098230 PMC8190486

[37] Malone JC Arbor TC Shah AB. Embryology, Midgut. In StatPearls [Internet]. Treasure Island, FL: StatPearls Publishing. (2025). 31985949

[38] De Renzi G Gaballo G Gazzaniga P Nicolazzo C. Molecular biomarkers according to primary tumor location in colorectal cancer: current standard and new insights. Oncology. (2021) 99:135–43. doi: 10.1159/000510944 33130682

[39] Han Y Liu D Li L. PD-1/PD-L1 pathway: current researches in cancer. Am J Cancer Res. (2020) 10:727–42. PMC7136921 32266087

[40] Ju X Zhang H Zhou Z Wang Q. Regulation of pd-L1 expression in cancer and clinical implications in immunotherapy. Am J Cancer Res. (2020) 10:1–11. 32064150 PMC7017746

[41] Lou E Xiu J Baca Y Nelson AC Weinberg BA Beg MS. Expression of immuno-oncologic biomarkers is enriched in colorectal cancers and other solid tumors harboring the A59t variant of kras. Cells. (2021) 10(6):1275. doi: 10.3390/cells10061275 34063999 PMC8224072

[42] Liao W Overman MJ Boutin AT Shang X Zhao D Dey P. Kras-irf2 axis drives immune suppression and immune therapy resistance in colorectal cancer. Cancer Cell. (2019) 35:559–72 e7. doi: 10.1016/j.ccell.2019.02.008 30905761 PMC6467776

[43] Xu M Zhao X Wen T Qu X. Unveiling the role of kras in tumor immune microenvironment. BioMed Pharmacother. (2024) 171:116058. doi: 10.1016/j.biopha.2023.116058 38171240

[44] Dong S Guo X Han F He Z Wang Y. Emerging role of natural products in cancer immunotherapy. Acta Pharm Sin B. (2022) 12:1163–85. doi: 10.1016/j.apsb.2021.08.020 PMC9069318 35530162

[45] Wang L Lynch C Pitroda SP Piffko A Yang K Huser AK. Radiotherapy and immunology. J Exp Med. (2024) 221(7):e20232101. doi: 10.1084/jem.20232101 38771260 PMC11110906

[46] Aktar S Hamid FB Gamage SMK Cheng T Parkneshan N Lu CT. Gene expression analysis of immune regulatory genes in circulating tumour cells and peripheral blood mononuclear cells in patients with colorectal carcinoma. Int J Mol Sci. (2023) 24(5):5051. doi: 10.3390/ijms24055051 36902476 PMC10003441

[47] He Y Zhu M Lai X Zhang H Jiang W. The roles of pd-L1 in the various stages of tumor metastasis. Cancer Metastasis Rev. (2024) 43:1475–88. doi: 10.1007/s10555-024-10189-4 38733457

[48] Wang NH Lei Z Yang HN Tang Z Yang MQ Wang Y. Radiation-induced pd-L1 expression in tumor and its microenvironment facilitates cancer-immune escape: A narrative review. Ann Transl Med. (2022) 10:1406. doi: 10.21037/atm-22-6049 36660640 PMC9843429

[49] Malkawi W Lutfi A Afghan MK Shah LM Costandy L Ramirez AB. Circulating tumour cell enumeration, biomarker analyses, and kinetics in patients with colorectal cancer and other gi Malignancies. Front Oncol. (2023) 13:1305181. doi: 10.3389/fonc.2023.1305181 38044994 PMC10693413

[50] Li L Yu D Yang J Zhang F Zhang D Lin Z. Significant response to pembrolizumab plus lenvatinib in epstein-barr-virus-associated intrahepatic cholangiocarcinoma: A case report. Cancer Biol Ther. (2024) 25:2338644. doi: 10.1080/15384047.2024.2338644 38650446 PMC11042061

[51] Yue C Jiang Y Li P Wang Y Xue J Li N. Dynamic change of pd-L1 expression on circulating tumor cells in advanced solid tumor patients undergoing pd-1 blockade therapy. Oncoimmunology. (2018) 7:e1438111. doi: 10.1080/2162402X.2018.1438111 29900038 PMC5993493

[52] Tan Z Yue C Ji S Zhao C Jia R Zhang Y. Assessment of pd-L1 expression on circulating tumor cells for predicting clinical outcomes in patients with cancer receiving pd-1/pd-L1 blockade therapies. Oncologist. (2021) 26:e2227–e38. doi: 10.1002/onco.13981 PMC8649012 34516729

[53] Raimondi L Raimondi FM Di Benedetto L Cimino G Spinelli GP. Pd-L1 expression on circulating tumour cells may be predictive of response to regorafenib in patients diagnosed with chemorefractory metastatic colorectal cancer. Int J Mol Sci. (2020) 21(18):6907. doi: 10.3390/ijms21186907 32962309 PMC7555209

[54] Sun B Chen H Wang X Chen T. Regorafenib induces bim-mediated intrinsic apoptosis by blocking akt-mediated foxo3a nuclear export. Cell Death Discov. (2023) 9:37. doi: 10.1038/s41420-023-01338-9 36720853 PMC9889785

[55] Dirven I Pierre E Vander Mijnsbrugge AS Vounckx M Kessels JI Neyns B. Regorafenib combined with braf/mek inhibitors for the treatment of refractory melanoma brain metastases. Cancers (Basel). (2024) 16(23):4083. doi: 10.3390/cancers16234083 39682270 PMC11640054

[56] Okpalanwaka IF Daugherity EA McCormick AL Anderson TS Smith SL Lawrence C. A pd-L1/cd3 bispecific antibody enhances the anti-tumor effects of regorafenib against colon cancer. Mol Cancer Ther. (2025). doi: 10.1158/1535-7163.MCT-24-1015 40202172

[57] Rzhevskiy A Kapitannikova A Malinina P Volovetsky A Aboulkheyr Es H Kulasinghe A. Emerging role of circulating tumor cells in immunotherapy. Theranostics. (2021) 11:8057–75. doi: 10.7150/thno.59677 PMC8315079 34335980

[58] Steinert G Scholch S Niemietz T Iwata N Garcia SA Behrens B. Immune escape and survival mechanisms in circulating tumor cells of colorectal cancer. Cancer Res. (2014) 74:1694–704. doi: 10.1158/0008-5472.CAN-13-1885 24599131

[59] Shasha T Gruijs M van Egmond M. Mechanisms of colorectal liver metastasis development. Cell Mol Life Sci. (2022) 79:607. doi: 10.1007/s00018-022-04630-6 36436127 PMC9701652

[60] Lin A Zhang J Luo P. Crosstalk between the msi status and tumor microenvironment in colorectal cancer. Front Immunol. (2020) 11:2039. doi: 10.3389/fimmu.2020.02039 32903444 PMC7435056

[61] Jain N Srinivasarao DA Famta P Shah S Vambhurkar G Shahrukh S. The portrayal of macrophages as tools and targets: A paradigm shift in cancer management. Life Sci. (2023) 316:121399. doi: 10.1016/j.lfs.2023.121399 36646378

[62] El-Kenawi A Hanggi K Ruffell B. The immune microenvironment and cancer metastasis. Cold Spring Harb Perspect Med. (2020) 10(4):a037424. doi: 10.1101/cshperspect.a037424 31501262 PMC7117953

[63] Inagaki K Kunisho S Takigawa H Yuge R Oka S Tanaka S. Role of tumor-associated macrophages at the invasive front in human colorectal cancer progression. Cancer Sci. (2021) 112:2692–704. doi: 10.1111/cas.14940 PMC8253270 33964093

[64] Yunna C Mengru H Lei W Weidong C. Macrophage M1/M2 polarization. Eur J Pharmacol. (2020) 877:173090. doi: 10.1016/j.ejphar.2020.173090 32234529

[65] Karlsson S Nystrom H. The extracellular matrix in colorectal cancer and its metastatic settling - alterations and biological implications. Crit Rev Oncol Hematol. (2022) 175:103712. doi: 10.1016/j.critrevonc.2022.103712 35588938

[66] Dietz MS Sutton TL Walker BS Gast CE Zarour L Sengupta SK. Relevance of circulating hybrid cells as a non-invasive biomarker for myriad solid tumors. Sci Rep. (2021) 11:13630. doi: 10.1038/s41598-021-93053-7 34211050 PMC8249418

[67] Manjunath Y Porciani D Mitchem JB Suvilesh KN Avella DM Kimchi ET. Tumor-cell-macrophage fusion cells as liquid biomarkers and tumor enhancers in cancer. Int J Mol Sci. (2020) 21(5):1872. doi: 10.3390/ijms21051872 32182935 PMC7084898

[68] Wei C Yang C Wang S Shi D Zhang C Lin X. Crosstalk between cancer cells and tumor associated macrophages is required for mesenchymal circulating tumor cell-mediated colorectal cancer metastasis. Mol Cancer. (2019) 18:64. doi: 10.1186/s12943-019-0976-4 30927925 PMC6441214

[69] Friedman-DeLuca M Karagiannis GS Condeelis JS Oktay MH Entenberg D. Macrophages in tumor cell migration and metastasis. Front Immunol. (2024) 15:1494462. doi: 10.3389/fimmu.2024.1494462 39555068 PMC11563815

[70] Magri V De Renzi G Marino L De Meo M Siringo M Gelibter A. Circulating cancer-associated macrophage-like cells as a blood-based biomarker of response to immune checkpoint inhibitors. Int J Mol Sci. (2024) 25(7):3752. doi: 10.3390/ijms25073752 38612563 PMC11011814

[71] Ren J He J Zhang H Xia Y Hu Z Loughran P. Platelet tlr4-erk5 axis facilitates net-mediated capturing of circulating tumor cells and distant metastasis after surgical stress. Cancer Res. (2021) 81:2373–85. doi: 10.1158/0008-5472.CAN-20-3222 PMC8137664 33687949

[72] Pereira-Veiga T Schneegans S Pantel K Wikman H. Circulating tumor cell-blood cell crosstalk: biology and clinical relevance. Cell Rep. (2022) 40:111298. doi: 10.1016/j.celrep.2022.111298 36044866

[73] Hu C Long L Lou J Leng M Yang Q Xu X. Ctc-neutrophil interaction: A key driver and therapeutic target of cancer metastasis. BioMed Pharmacother. (2024) 180:117474. doi: 10.1016/j.biopha.2024.117474 39316968

[74] Mizuno R Kawada K Itatani Y Ogawa R Kiyasu Y Sakai Y. The role of tumor-associated neutrophils in colorectal cancer. Int J Mol Sci. (2019) 20(3):529. doi: 10.3390/ijms20030529 30691207 PMC6386937

[75] Saini M Szczerba BM Aceto N. Circulating tumor cell-neutrophil tango along the metastatic process. Cancer Res. (2019) 79:6067–73. doi: 10.1158/0008-5472.CAN-19-1972 31527091

[76] Garrido-Navas C de Miguel-Perez D Exposito-Hernandez J Bayarri C Amezcua V Ortigosa A. Cooperative and escaping mechanisms between circulating tumor cells and blood constituents. Cells. (2019) 8(11):1382. doi: 10.3390/cells8111382 31684193 PMC6912439

[77] Sionov RV. Leveling up the controversial role of neutrophils in cancer: when the complexity becomes entangled. Cells. (2021) 10(9):2486. doi: 10.3390/cells10092486 34572138 PMC8465406

[78] Fatima S Ma Y Safrachi A Haider S Spring KJ Vafaee F. Harnessing liquid biopsies to guide immune checkpoint inhibitor therapy. Cancers (Basel). (2022) 14(7):1669. doi: 10.3390/cancers14071669 35406441 PMC8997025

[79] Liu X Song J Zhang H Liu X Zuo F Zhao Y. Immune checkpoint hla-E:Cd94-nkg2a mediates evasion of circulating tumor cells from nk cell surveillance. Cancer Cell. (2023) 41:272–87 e9. doi: 10.1016/j.ccell.2023.01.001 36706761

[80] Yi Y Qin G Yang H Jia H Zeng Q Zheng D. Mesenchymal stromal cells increase the natural killer resistance of circulating tumor cells via intercellular signaling of cgas-sting-ifnbeta-hla. Adv Sci (Weinh). (2024) 11:e2400888. doi: 10.1002/advs.202400888 38638003 PMC11151078

[81] Yang C Wang X To KKW Cui C Luo M Wu S. Circulating tumor cells shielded with extracellular vesicle-derived cd45 evade T cell attack to enable metastasis. Signal Transduct Target Ther. (2024) 9:84. doi: 10.1038/s41392-024-01789-1 38575583 PMC10995208

[82] Ma Y Wei J He W Ren J. Neutrophil extracellular traps in cancer. MedComm (2020). (2024) 5:e647. doi: 10.1002/mco2.647 39015554 PMC11247337

[83] Bao Y Zhang D Guo H Ma W. Beyond blood: advancing the frontiers of liquid biopsy in oncology and personalized medicine. Cancer Sci. (2024) 115:1060–72. doi: 10.1111/cas.16097 PMC11007055 38308498

[84] Zhu G Pei L Xia H Tang Q Bi F. Role of oncogenic kras in the prognosis, diagnosis and treatment of colorectal cancer. Mol Cancer. (2021) 20:143. doi: 10.1186/s12943-021-01441-4 34742312 PMC8571891

[85] Grothey A Fakih M Tabernero J. Management of braf-mutant metastatic colorectal cancer: A review of treatment options and evidence-based guidelines. Ann Oncol. (2021) 32:959–67. doi: 10.1016/j.annonc.2021.03.206 33836264

[86] Ciombor KK Strickler JH Bekaii-Saab TS Yaeger R. Braf-mutated advanced colorectal cancer: A rapidly changing therapeutic landscape. J Clin Oncol. (2022) 40:2706–15. doi: 10.1200/JCO.21.02541 PMC9390817 35649231

[87] Yu B Ma W. Biomarker discovery in hepatocellular carcinoma (Hcc) for personalized treatment and enhanced prognosis. Cytokine Growth Factor Rev. (2024) 79:29–38. doi: 10.1016/j.cytogfr.2024.08.006 39191624

[88] Denis JA Patroni A Guillerm E Pepin D Benali-Furet N Wechsler J. Droplet digital pcr of circulating tumor cells from colorectal cancer patients can predict kras mutations before surgery. Mol Oncol. (2016) 10:1221–31. doi: 10.1016/j.molonc.2016.05.009 PMC5423194 27311775

[89] Damin F Galbiati S Soriani N Burgio V Ronzoni M Ferrari M. Analysis of kras, nras and braf mutational profile by combination of in-tube hybridization and universal tag-microarray in tumor tissue and plasma of colorectal cancer patients. PloS One. (2018) 13:e0207876. doi: 10.1371/journal.pone.0207876 30562355 PMC6298683

[90] Saadi S Aarab M Tabyaoui I Jouti NT. Circulating tumor cells in colorectal cancer - a review of detection methods and clinical relevance. Contemp Oncol (Pozn). (2023) 27:123–31. doi: 10.5114/wo.2023.133740 PMC10793619 38239860

[91] Musina AM Zlei M Mentel M Scripcariu DV Stefan M Anitei MG. Evaluation of circulating tumor cells in colorectal cancer using flow cytometry. J Int Med Res. (2021) 49:300060520980215. doi: 10.1177/0300060520980215 34587798 PMC8489760

[92] Ferreira JA Magalhaes A Gomes J Peixoto A Gaiteiro C Fernandes E. Protein glycosylation in gastric and colorectal cancers: toward cancer detection and targeted therapeutics. Cancer Lett. (2017) 387:32–45. doi: 10.1016/j.canlet.2016.01.044 26828132

[93] Lima L Neves M Oliveira MI Dieguez L Freitas R Azevedo R. Sialyl-tn identifies muscle-invasive bladder cancer basal and luminal subtypes facing decreased survival, being expressed by circulating tumor cells and metastases. Urol Oncol. (2017) 35:675 e1– e8. doi: 10.1016/j.urolonc.2017.08.012 28911924

[94] Neves M Azevedo R Lima L Oliveira MI Peixoto A Ferreira D. Exploring sialyl-tn expression in microfluidic-isolated circulating tumour cells: A novel biomarker and an analytical tool for precision oncology applications. N Biotechnol. (2019) 49:77–87. doi: 10.1016/j.nbt.2018.09.004 30273682

[95] Yao S Han Y Yang M Jin K Lan H. Integration of liquid biopsy and immunotherapy: opening a new era in colorectal cancer treatment. Front Immunol. (2023) 14:1292861. doi: 10.3389/fimmu.2023.1292861 38077354 PMC10702507

[96] Rushton AJ Nteliopoulos G Shaw JA Coombes RC. A review of circulating tumour cell enrichment technologies. Cancers (Basel). (2021) 13(5):970. doi: 10.3390/cancers13050970 33652649 PMC7956528

[97] Panda SS Lee CC Geevimaan K Chen KC Yang SH Shen CN. Intracellular domain of epithelial cell adhesion molecule induces wnt receptor transcription to promote colorectal cancer progression. J BioMed Sci. (2024) 31:72. doi: 10.1186/s12929-024-01057-y 39010070 PMC11247908

[98] Chiu SY Hsieh CH You JF Chu PY Hung HY Chu PH. Enhancing prediction performance by add-on combining circulating tumor cell count, cd45(Neg) epcam(Neg) cell count on colorectal cancer, advance, and metastasis. Cancers (Basel). (2021) 13(11):2521. doi: 10.3390/cancers13112521 34063929 PMC8196640

[99] Lu X Tan S Wu M Ju H Liang X Li P. Evaluation of a new magnetic bead as an integrated platform for systematic ctc recognition, capture and clinical analysis. Colloids Surf B Biointerfaces. (2021) 199:111542. doi: 10.1016/j.colsurfb.2020.111542 33373845

[100] Ma S Zhou M Xu Y Gu X Zou M Abudushalamu G. Clinical application and detection techniques of liquid biopsy in gastric cancer. Mol Cancer. (2023) 22:7. doi: 10.1186/s12943-023-01715-z 36627698 PMC9832643

[101] Alves JM Estevez-Gomez N Valecha M Prado-Lopez S Tomas L Alvarino P. Comparative analysis of capture methods for genomic profiling of circulating tumor cells in colorectal cancer. Genomics. (2022) 114:110500. doi: 10.1016/j.ygeno.2022.110500 36202322

[102] Lin S Feng D Han X Li L Lin Y Gao H. Microfluidic platform for omics analysis on single cells with diverse morphology and size: A review. Anal Chim Acta. (2024) 1294:342217. doi: 10.1016/j.aca.2024.342217 38336406

[103] Bhat MP Thendral V Uthappa UT Lee KH Kigga M Altalhi T. Recent advances in microfluidic platform for physical and immunological detection and capture of circulating tumor cells. Biosensors (Basel). (2022) 12(4):220. doi: 10.3390/bios12040220 35448280 PMC9025399

[104] Yang C Chen F Wang S Xiong B. Circulating tumor cells in gastrointestinal cancers: current status and future perspectives. Front Oncol. (2019) 9:1427. doi: 10.3389/fonc.2019.01427 31921680 PMC6923205

[105] Chu HY Lu LS Cho W Wu SY Chang YC Lin CP. Enumerating circulating tumor cells with a self-assembled cell array (Saca) chip: A feasibility study in patients with colorectal cancer. Cancers (Basel). (2019) 11(1):56. doi: 10.3390/cancers11010056 30626171 PMC6356678

[106] Carneiro A Piairo P Teixeira A Ferreira D Cotton S Rodrigues C. Discriminating epithelial to mesenchymal transition phenotypes in circulating tumor cells isolated from advanced gastrointestinal cancer patients. Cells. (2022) 11(3):376. doi: 10.3390/cells11030376 35159186 PMC8834092

[107] Sun H Hu N Wang J. Application of microfluidic technology in antibody screening. Biotechnol J. (2022) 17:e2100623. doi: 10.1002/biot.202100623 35481726

[108] Li W Wu CC Wang S Zhou L Qiao L Ba W. Identification of the target protein of the metastatic colorectal cancer-specific aptamer W3 as a biomarker by aptamer-based target cells sorting and functional characterization. Biosens Bioelectron. (2022) 213:114451. doi: 10.1016/j.bios.2022.114451 35700603

[109] Moutsiopoulou A Broyles D Dikici E Daunert S Deo SK. Molecular aptamer beacons and their applications in sensing, imaging, and diagnostics. Small. (2019) 15:e1902248. doi: 10.1002/smll.201902248 31313884 PMC6715520

[110] Lu Y Li X Liu Y Li J Chen Z Meng X. Novel molecular aptamer beacon for the specific simultaneous analysis of circulating tumor cells and exosomes of colorectal cancer patients. Anal Chem. (2023) 95:1251–61. doi: 10.1021/acs.analchem.2c04017 36583760

[111] Chen Y Yang Y Feng J Carrier AJ Tyagi D Yu X. A universal monoclonal antibody-aptamer conjugation strategy for selective non-invasive bioparticle isolation from blood on a regenerative microfluidic platform. Acta Biomater. (2022) 152:210–20. doi: 10.1016/j.actbio.2022.09.001 36087870

[112] Jia F Wang Y Fang Z Dong J Shi F Zhang W. Novel peptide-based magnetic nanoparticle for mesenchymal circulating tumor cells detection. Anal Chem. (2021) 93:5670–5. doi: 10.1021/acs.analchem.1c00577 33788544

[113] Chowdhury T Cressiot B Parisi C Smolyakov G Thiebot B Trichet L. Circulating tumor cells in cancer diagnostics and prognostics by single-molecule and single-cell characterization. ACS Sens. (2023) 8:406–26. doi: 10.1021/acssensors.2c02308 36696289

[114] Farkas B de Leeuw NH. A perspective on modelling metallic magnetic nanoparticles in biomedicine: from monometals to nanoalloys and ligand-protected particles. . Materials (Basel). (2021) 14(13):3611. doi: 10.3390/ma14133611 34203371 PMC8269646

[115] Yun Y Kim S Lee SN Cho HY Choi JW. Nanomaterial-based detection of circulating tumor cells and circulating cancer stem cells for cancer immunotherapy. Nano Converg. (2024) 11:56. doi: 10.1186/s40580-024-00466-x 39671082 PMC11645384

[116] Nayl AA Abd-Elhamid AI Aly AA Brase S. Recent progress in the applications of silica-based nanoparticles. RSC Adv. (2022) 12:13706–26. doi: 10.1039/d2ra01587k PMC9073631 35530394

[117] Kefayat A Sartipzadeh O Molaabasi F Amiri M Gholami R Mirzadeh M. Microfluidic system consisting of a magnetic 3d-printed microchannel filter for isolation and enrichment of circulating tumor cells targeted by anti-her2/mof @Ferrite core-shell nanostructures: A theranostic ctc dialysis system. Anal Chem. (2024) 96:4377–84. doi: 10.1021/acs.analchem.3c03567 38442207

[118] Li Z Qin C Zhao B Li T Zhao Y Zhang X. Circulating tumor cells in pancreatic cancer: more than liquid biopsy. Ther Adv Med Oncol. (2024) 16:17588359241284935. doi: 10.1177/17588359241284935 39421679 PMC11483845

[119] Mjahed RB Astaras C Roth A Koessler T. Where are we now and where might we be headed in understanding and managing brain metastases in colorectal cancer patients? Curr Treat Options Oncol. (2022) 23:980–1000. doi: 10.1007/s11864-022-00982-0 35482170 PMC9174111

[120] Ko SW Yoon SB. Clinical implications and perspectives of portal venous circulating tumor cells in pancreatic cancer. World J Gastrointest Oncol. (2023) 15:632–43. doi: 10.4251/wjgo.v15.i4.632 PMC10134213 37123055

[121] Kilgour E Rothwell DG Brady G Dive C. Liquid biopsy-based biomarkers of treatment response and resistance. Cancer Cell. (2020) 37:485–95. doi: 10.1016/j.ccell.2020.03.012 32289272

[122] Gruijs M Zeelen C Hellingman T Smit J Borm FJ Kazemier G. Detection of circulating tumor cells using the attune nxt. Int J Mol Sci. (2022) 24(1):21. doi: 10.3390/ijms24010021 36613466 PMC9820284

[123] Xu J Liao K Yang X Wu C Wu W. Using single-cell sequencing technology to detect circulating tumor cells in solid tumors. Mol Cancer. (2021) 20:104. doi: 10.1186/s12943-021-01392-w 34412644 PMC8375060

[124] Ho HY Chung KK Kan CM Wong SC. Liquid biopsy in the clinical management of cancers. Int J Mol Sci. (2024) 25(16):8594. doi: 10.3390/ijms25168594 39201281 PMC11354853

[125] Lopresti A Malergue F Bertucci F Liberatoscioli ML Garnier S DaCosta Q. Sensitive and easy screening for circulating tumor cells by flow cytometry. JCI Insight. (2019) 5(14):e128180. doi: 10.1172/jci.insight.128180 31194699 PMC6675556

[126] Pei H Li L Wang Y Sheng R Wang Y Xie S. Single-cell phenotypic profiling of ctcs in whole blood using an integrated microfluidic device. Anal Chem. (2019) 91:11078–84. doi: 10.1021/acs.analchem.9b01647 31373191

[127] Ai J Huang Y Yin Z Deng Y Yan L Liao J. Sea anemone-inspired conducting polymer sensing platform for integrated detection of tumor protein marker and circulating tumor cell. Adv Healthc Mater. (2024) 13:e2401305. doi: 10.1002/adhm.202401305 38767216

[128] Francescangeli F Magri V De Angelis ML De Renzi G Gandini O Zeuner A. Sequential isolation and characterization of single ctcs and large ctc clusters in metastatic colorectal cancer patients. Cancers (Basel). (2021) 13(24):6362. doi: 10.3390/cancers13246362 34944983 PMC8699456

[129] Yu B Shao S Ma W. Frontiers in pancreatic cancer on biomarkers, microenvironment, and immunotherapy. Cancer Lett. (2025) 610:217350. doi: 10.1016/j.canlet.2024.217350 39581219

[130] Liu Y Zugazagoitia J Ahmed FS Henick BS Gettinger SN Herbst RS. Immune cell pd-L1 colocalizes with macrophages and is associated with outcome in pd-1 pathway blockade therapy. Clin Cancer Res. (2020) 26:970–7. doi: 10.1158/1078-0432.CCR-19-1040 PMC7024671 31615933

[131] Zhang P Draz MS Xiong A Yan W Han H Chen W. Immunoengineered magnetic-quantum dot nanobead system for the isolation and detection of circulating tumor cells. J Nanobiotechnology. (2021) 19:116. doi: 10.1186/s12951-021-00860-1 33892737 PMC8063296

[132] Wu CY Fu JY Wu CF Hsieh MJ Liu YH Liu HP. Malignancy prediction capacity and possible prediction model of circulating tumor cells for suspicious pulmonary lesions. J Pers Med. (2021) 11(6):444. doi: 10.3390/jpm11060444 34064011 PMC8223995

[133] Aranda E Vieitez JM Gomez-Espana A Gil Calle S Salud-Salvia A Grana B. Folfoxiri plus bevacizumab versus folfox plus bevacizumab for patients with metastatic colorectal cancer and >/=3 circulating tumour cells: the randomised phase iii visnu-1 trial. ESMO Open. (2020) 5:e000944. doi: 10.1136/esmoopen-2020-000944 33148620 PMC7640586

[134] Hao YJ Chang LW Yang CY Lo LC Lin CP Jian YW. The rare circulating tumor microemboli as a biomarker contributes to predicting early colorectal cancer recurrences after medical treatment. Transl Res. (2024) 263:1–14. doi: 10.1016/j.trsl.2023.07.011 37558203

[135] Crisafulli G. Liquid biopsy and challenge of assay heterogeneity for minimal residual disease assessment in colon cancer treatment. Genes (Basel). (2025) 16(1):71. doi: 10.3390/genes16010071 39858618 PMC11765229

[136] Sastre J Orden V Martinez A Bando I Balbin M Bellosillo B. Association between baseline circulating tumor cells, molecular tumor profiling, and clinical characteristics in a large cohort of chemo-naive metastatic colorectal cancer patients prospectively collected. Clin Colorectal Cancer. (2020) 19:e110–e6. doi: 10.1016/j.clcc.2020.02.014 32278676

[137] Abdallah EA Souza ESV Braun AC Gasparini VA Kupper BEC Tariki MS. A higher platelet-to-lymphocyte ratio is prevalent in the presence of circulating tumor microemboli and is a potential prognostic factor for non-metastatic colon cancer. Transl Oncol. (2021) 14:100932. doi: 10.1016/j.tranon.2020.100932 33157516 PMC7649529

[138] Tieng FYF Abu N Lee LH Ab Mutalib NS. Microsatellite instability in colorectal cancer liquid biopsy-current updates on its potential in non-invasive detection, prognosis and as a predictive marker. Diagnostics (Basel). (2021) 11(3):544. doi: 10.3390/diagnostics11030544 33803882 PMC8003257

[139] van ‘t Erve I Medina JE Leal A Papp E Phallen J Adleff V. Metastatic colorectal cancer treatment response evaluation by ultra-deep sequencing of cell-free DNA and matched white blood cells. Clin Cancer Res. (2023) 29:899–909. doi: 10.1158/1078-0432.CCR-22-2538 36534496 PMC9975664

[140] Bidard FC Kiavue N Ychou M Cabel L Stern MH Madic J. Circulating tumor cells and circulating tumor DNA detection in potentially resectable metastatic colorectal cancer: A prospective ancillary study to the unicancer prodige-14 trial. Cells. (2019) 8(6):516. doi: 10.3390/cells8060516 31142037 PMC6627974

